# Association between serum lipid concentrations and attempted suicide in patients with major depressive disorder: A meta-analysis

**DOI:** 10.1371/journal.pone.0243847

**Published:** 2020-12-10

**Authors:** Huan Li, Xueyan Zhang, Qing Sun, Rui Zou, Zhijun Li, Shengyuan Liu

**Affiliations:** 1 School of Public Health, Beihua University, Jilin, Jilin Province, China; 2 School of Nursing, Jilin University, Changchun, Jilin Province, China; 3 Department of Clinical Nutrition, Affiliated Hospital of Jilin Medical University, Jilin, Jilin Province, China; 4 Shenzhen Nanshan Center for Chronic Disease Control, Shenzhen, Guangdong Province, China; Chiba Daigaku, JAPAN

## Abstract

**Background:**

There is growing evidence that serum lipid concentrations may be associated with attempted suicide in patients with major depressive disorder (MDD), but these findings remain controversial. Thus, we performed a comprehensive meta-analysis to quantitatively assess the associations between serum lipid concentrations and attempted suicide in MDD patients.

**Materials and methods:**

Electronic databases (PubMed, Embase, the Cochrane Library and the China National Knowledge Library) were searched for relevant literature up to 10 February 2020. We used a random-effects model based on heterogeneity amongst studies and generated pooled standardised mean differences (SMDs).

**Results:**

Thirty-two studies comprising 7,068 subjects met the inclusion criteria. A pooled analysis showed that compared with non-attempters, MDD patients who had attempted suicide had significantly lower serum concentrations of total cholesterol (TC) (SMD: -0.63, 95% CI: -0.83 to -0.44) and low-density lipoprotein cholesterol (LDL-C) (SMD: -0.69, 95% CI: -1.04 to -0.34), but the serum concentrations of high-density lipoprotein cholesterol (HDL-C) (SMD: -0.12, 95% CI: -0.33 to 0.10) and triglycerides (TGs) (SMD: 0.00, 95% CI: -0.20 to 0.20) were not significantly different between the two groups. Subgroup and meta-regression analysis indicated that heterogeneity with respect to TC concentrations may be due to different ages (*p* = 0.041) and sample sizes (*p* = 0.016) of studies, and that heterogeneity with respect to HDL-C concentrations may be partly due to different settings of studies (*p* = 0.017).

**Conclusions:**

This meta-analysis demonstrated that lower concentrations of TC and LDL-C, but not of HDL-C and TGs, were associated with attempted suicide in MDD patients. This indicates that TC and LDL-C may be useful as biological markers for predicting whether MDD patients may attempt to commit suicide.

## Introduction

Major depressive disorder (MDD) is a common, recurrent, and chronic psychiatric disorder and is considered to be a major worldwide public-health problem. Data from the Global Burden of Diseases, Injuries, and Risk Factors Study 2016 indicated that MDD was the fifth leading cause of years-lived-with-disability (YLDs) worldwide in 2016, comprising 34.1 million (range, 23.5–46.0 million [4.2%, 3.2–5.3]) of total YLDs [1]. The worst outcome of MDD is suicide, and approximately 45%–75% of those who commit suicide have MDD [2, 3]. Thus, identifying predictors of suicide in MDD patients is particularly important.

Many studies have been performed to identify peripheral biological markers (e.g. serum lipids) that may be associated with attempted suicide and MDD, and which may be useful as predictive tools, although the neurobiological mechanisms of such associations are yet to be fully understood [4, 5]. The main neurochemical hypothesis is that there is an association between low cholesterol concentrations, poor serotonin uptake and a decrease in the viscosity of brain-cell membranes, and experimental evidence has confirmed that lipid fluidity markedly modulates the binding of serotonin in mouse brain membranes [3, 6].

Previous epidemiological studies have also assessed the associations between serum lipid concentrations and attempted suicide among MDD patients, but the results were conflicting. Some studies have suggested that low serum lipid concentrations were associated with increased suicide risk among MDD patients [3, 7–10], while others found that MDD patients who attempted suicide had higher serum lipid concentrations than non-attempters [11, 12], or that there was no difference in serum lipid concentrations between the two groups of MDD patients [13–15]. In 2017, a meta-analysis by Wu et al. [16] found an inverse association between serum lipid concentrations and suicidality in patients with various psychiatric diseases, including depression, schizophrenia, personality disorder, and drug and alcohol addictions. However, people with various depressive disorders (depression, schizoaffective depression, major depressive episodes and MDD) in their study had been combined into “depression group” and analysed together. Therefore, studies that have comprehensively pooled evidence on the association between serum lipid concentrations and attempted suicide only in MDD patients remained sparse. In addition, although there is growing evidence from epidemiological studies of such an association [17–22], this has yet to be clarified and validated.

Thus, we performed an updated systematic review and meta-analysis to quantitatively assess the association between serum lipid concentrations and attempted suicide in MDD patients.

## Materials and methods

### Search strategy and study selection

We comprehensively searched electronic databases, namely PubMed, Embase, Cochrane Library and the China National Knowledge Library, for potentially eligible literature, from inception to 10 February 2020. Studies were identified using a combination of the following search terms: (‘major depressive disorder’ or ‘depression’) and ‘suicide’ and (‘cholesterol’ or ‘high-density lipoprotein’ or ‘HDL’ or ‘low-density lipoprotein’ or ‘LDL’ or ‘triglycerides’ or ‘lipid*’), without restrictions on language or study design. We also manually checked the reference lists of retrieved studies and review articles to identify any relevant studies that were missed in the database searches. We attempted to contact the authors of any studies that lacked information. We performed our meta-analysis in accordance with the Meta-analyses Of Observational Studies in Epidemiology guidelines [23].

### Inclusion criteria

A study was included in this meta-analysis if it met the following criteria: (1) assessed at least one of the following serum lipid biomarkers: total cholesterol (TC), high-density lipoprotein cholesterol (HDL-C), low-density lipoprotein cholesterol (LDL-C) or triglyceride (TG) concentrations; (2) had suicide attempt as the outcome, which was defined by a subject exhibiting self-injurious behaviours, with suicidal ideation, a suicide plan and suicidal tendencies; (3) was conducted in patients with diagnosed MDD; and (4) reported means ± SD (standard deviation) of lipid concentrations or sufficient information for these to be calculated. We excluded reviews, animal and mechanistic studies, and conference abstracts. If studies had overlapping subjects, only the latest or more comprehensive study was included. The selection of studies and data extraction were independently performed by two investigators (LH and ZX), and any uncertainties or disagreements were resolved by discussions among all authors to reach consensus.

### Data extraction and quality assessment

Two authors independently extracted the relevant information of all eligible studies using a data-extraction form. The following items were extracted from each study, if available: author name(s), publication year, country of study, sex of participants, mean age of participants, lipid concentrations of participants, study setting, sample type, sample size, number of MDD patients who attempted suicide, and suicide time-frame.

Two authors independently evaluated the quality of all of the studies, based on the Newcastle-Ottawa Scale [24]. Cohort and case-control studies with eight or more stars and cross-sectional studies with four or more stars were regarded as relatively high quality; otherwise, studies were regarded as relatively low quality.

### Statistical analysis

We investigated the associations between the lipid biomarkers (i.e. TC, HDL-C, LDL-C, and TGs) and the risk of stroke as the main analyses. For TC, LDL-C, and HDL-C, 1 mmol/L was converted to 38.66 mg/dL; for TGs, 1 mmol/L was converted to 88.60 mg/dL. For each biomarker, standardised mean differences (SMDs) and 95% confidence intervals (CIs) were used to estimate the effect sizes of the association between lipid concentrations and attempted suicide in MDD patients and were performed using a random-effects model based on the DerSimonian and Laird method [25]. The random-effects model was selected a priori because it is more conservative than a fixed-effects model, as it accounts for both within- and between-study heterogeneity [26]. *I*^2^ (higher values indicating greater heterogeneity) and Q tests were used to evaluate the heterogeneity among studies [27]. An *I*^2^ value of <25% denoted low heterogeneity, 25%–75% denoted moderate heterogeneity, and >75% denoted high heterogeneity [27].

Because significant heterogeneity was observed in the overall analyses, subgroup analyses were conducted to explore potential sources of heterogeneity, which were selected a priori, namely age (<40 vs. ≥40 years), geographical area (America, Asia, Europe, or Oceania), setting (inpatient, outpatient, or combined), sample size (<100 vs. ≥100), study design (case-control vs. cross-sectional), publication year (before 2007 vs. 2007 or later), suicidal time-frame (recent vs. lifetime), blood sample (fasting vs. non-fasting), treatment (yes vs. no) and study quality (relatively high vs. relatively low). Sensitivity analyses were performed by excluding one study at a time to evaluate the influence of each study on the overall estimate. In addition, meta-regression analyses were performed to investigate potential sources of between-study heterogeneity.

Visual inspection by funnel plots, Begg’s test and Egger’s test was used to assess the publication bias of pooled results [28, 29]. The number of missing studies and the effect of these studies on the overall effects were explored using a nonparametric rank-based data augmentation technique (trim-and-fill procedure) developed by Duval and Tweedie [30]. Stata software version 14.0 (Stata Corp.) was used for statistical analysis, and a two-sided *P* value of less than 0.05 was considered to indicate statistical significance in all analyses.

## Results

### Search results

**Fig 1:**
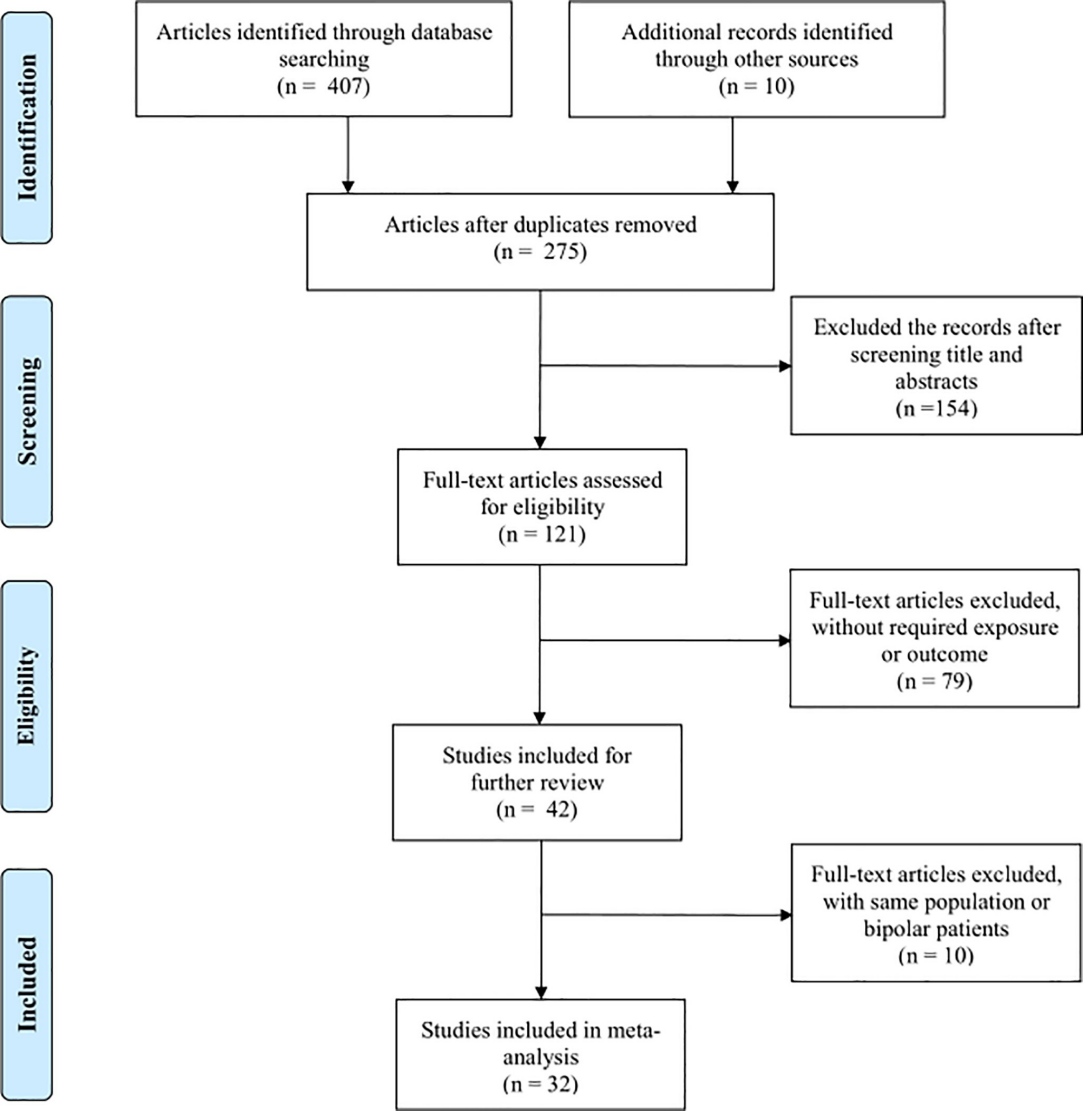
Summary of the eligible studies selection process.

Our search strategy generated 417 records. After removing duplicate citations and unrelated articles by screening titles and abstracts, we identified 121 potentially eligible studies for full-text screening. Then, a further 90 articles were excluded, due to their lack of the required exposure or outcome, being duplicates, or comprising bipolar subjects. Finally, 32 studies [3, 7, 9, 10, 13, 15, 17–21, 30–44], comprising a total of 7,068 participants, met the eligibility criteria and were included in our meta-analysis for assessing the associations between serum lipid concentrations and attempted suicide in MDD patients (Fig 1). A summary of the characteristics of the eligible studies is presented in Table 1.

**Table 1:** Characteristics of studies included in the meta-analysis.

Authors, year	Study design	Study country (area)	Gender	Age (years)	Setting	Lipid(s)	Time-frame to SA	SA (n, mean±sd, mg/dl)	NC (n, mean±sd, mg/dl)	Quality score
Ainiyet B et al, 2014	Cross-sectional	Polan (Europe)	73 male, 150 female	45±15 (male), 49±13 (female)	Inpatients	TC	Current	^a^10, 191±43 ^b^16, 213±50	^a^15, 256±49 ^b^56, 253±50	3
						HDL-C	Current	^a^10, 51±21 ^b^16, 52±10	^a^15, 51±17 ^b^56, 56±18	
						LDL-C	Current	^a^10, 117±39 ^b^16, 139±49	^a^15, 172±45 ^b^56, 170±43	
						TG	Current	^a^10, 113±61 ^b^16, 112±38	^a^15, 163±99 ^b^56, 146±98	
Almeida-Montes LG et al, 2000	Cross-sectional	Mexico (America)	8 male, 25 female	32.0±12.9(SA), 31.9±8.8(NSA)	Inpatients+Outpatients	TC	Current	18,176.1±35.2	15,176.8±22.2	3
						HDL-C	Current	18,38.7±9.9	15,37.5±9.3	
						LDL-C	Current	18,100±34.1	15,112.2±21.3	
						TG	Current	18,155.5±71.9	15,140±59	
Baek JH et al, 2014	Cross-sectional	Korea (Asia)	150 male, 405 female	35.5±15.9(SA-recent), 41.6±15.4(lifetime SA), 47.5±15.6(NSA)	Outpatients	TC	Current Lifetime	22,180±32.9 69,194.9±31.9	464,190.5±36.3 464,190.5±36.3	5
						HDL-C	Current Lifetime	22, 68.8±16.9 69,62.4±18.7	464,58.6±15.4 464,58.6±15.4	
						LDL-C	Current Lifetime	22,102.7±30 69,114.3±32.5	464,114±33 464,114±33	
						TG	Current Lifetime	22, 89.5±36.8 69,114.4±63.5	464,129.9±87.2 464,129.9±87.2	
Bartoli F et al.,2016	Cross-sectional	Italy (Europe)	105 male, 109 female	55±15.8(SA), 57.7±14.5(NSA)	Inpatients	TC	Current	66,174±45.7	111,193.9±42.6	8
						TG	Current	66,118.7±69.5	111,129.7±111.4	
Chen Y et al,2003	Cross-sectional	China (Asia)	NA	42.9±13.6	Inpatients	TC	Current	33, 152.7±36.7	34, 174.4±45.2	8
						HDL-C	Current	33, 48.7±10.8	34, 53.7±11.6	
						LDL-C	Current	33, 83.5±14.7	34, 108.6±13.1	
Deisenhammer EA et al,2004	Cross-sectional	Austra (Europe)	34 male, 58 female	43.9±11.7	Inpatients	TC	Lifetime	37,208.7±30.6	55, 201.1±36.2	4
						HDL-C	Lifetime	37, 49.3±17.8	55, 46.3±12.4	
						LDL-C	Lifetime	37,134.1±49.6	55, 136.7±34.1	
						TG	Lifetime	37,125.6±49.6	55, 147±87.1	
Eidan AJ et al.,2019	Case-control	Iraq (Asia)	40 male, 20 female	36.9±10.3(SA), 30.8±14.1(NSA)	Outpatients	TC	Current	22,155.6±8.6	38,172±7.7	8
						HDL-C	Current	22,52.4±14.1	38,50.3±12.2	
						LDL-C	Current	22,81.6±5.9	38,94.4±5.5	
						TG	Current	22,166.2±36.8	38,175.9±29.1	
Ekinci O et al.,2017	Case-control	Turkey (Asia)	42 male, 97 female	43.9±14.2(SA), 41.9±11.5(NSA)	Inpatients	TC	Current	37,159.9±73.1	102,179.9±44	8
						HDL-C	Current	37,42.8±1.5	102,44.5±0.9	
						LDL-C	Current	37,94.6±6.1	102,108.4±3.7	
						TG	Current	37,152.3±12.9	102,130.6±7.8	
Fu B et al,1999	Case-control	China (Asia)	26 male,18 female	43±14.2	Inpatients	TC	Current	17, 125.7±15.5 ^a^10,125.3±15.1 ^b^7,126.4±17.4	27, 151.9±28.6 ^a^16,153.1±32.9 ^b^11,149.6±22.8	6
Guo X et al,2006	Case-control	China (Asia)	40 male, 26female	42±11	Inpatients	TC	Lifetime	25, 145.4±29.0	41,174.7±31.7	7
						HDL-C	Lifetime	25, 39.1±10.1	41, 39.1±8.9	
						LDL-C	Lifetime	25, 89.7±32.5	41,113.3±29.4	
						TG	Lifetime	25, 101.9±55.8	41,152.4±115.2	
Huang TL et al,2005	Cross-sectional	China (Asia)	54 male, 114 female	31.4±8.5	Inpatients	TC	Current	21,175.6±25.5 ^a^6,175.2±21.8 ^b^15,175.8±27.5	88, 180±34.4 ^a^26,186±38.8 ^b^62,177.4±32.4	3
						HDL-C	Current	21,175.6±25.5 ^a^6,175.2±21.8 ^b^15,175.8±27.5	88, 180±34.4 ^a^26,186±38.8 ^b^62,177.4±32.4	
						LDL-C	Current	21,175.6±25.5 ^a^6,175.2±21.8 ^b^15,175.8±27.5	88, 180±34.4 ^a^26,186±38.8 ^b^62,177.4±32.4	
						TG	Current	21,91.6±56 ^a^6,63.5±15.3 ^b^15,102.9±62.6	88,107.8±59.4 ^a^26,128.8±71.8 ^b^62,99.1±51.5	
Kim YK et al,2002	Case-control	Korea (Asia)	243 male, 282 female	38.1±17(SA), 38.2±16.3(NSA)	Outpatients	TC	Current	147,147.4±36.1	147,180.7±37.1	8
Kim YK et al,2004	Case-control	Korea (Asia)	225 male, 324 female	40.5±18.1(SA), 42.0±16.5(NSA)	Outpatients	TC	Current	149,149.9±34.4	149,179.6±37.2	8
Koponen H et al,2015	Cross-sectional	Finland (Europe)	140 male, 312 female	51±10 (with SB), 51±10(NSB)	Outpatients	TC	Lifetime	^c^218,201.8±41.0	^c^ 230,192.1±37.9	5
						HDL-C	Lifetime	^c^218,59.1±17.4	^c^ 230,62.2±19.3	
						LDL-C	Lifetime	^c^218,124.1±38.7	^c^ 230,112.1±32.1	
						TG	Lifetime	^c^218,131.1±81.5	^c^ 230,114.3±68.2	
Luo T et al,2015	Case-control	China (Asia)	75 male, 205 female	50.57±2.32	Inpatients	TC	Lifetime	72, 168.6±35.6	68, 169.3±37.5	7
						HDL-C	Lifetime	72, 51.8±13.14	68, 56.8±15.5	
						LDL-C	Lifetime	72, 96.3±30.9	68, 93.9±29.8	
						TG	Lifetime	72, 124.1±62.0	68, 111.6±54.93	
Ma YJ et al,2019	Cross-sectional	China (Asia)	100 male, 188 female	39 (SA), 42(NSA)	Inpatients	TC	Current	58, 158.5±38.7	230, 166.2±46.4	5
						HDL-C	Current	58, 46.4±15.5	230, 50.3±15.5	
						LDL-C	Current	58, 92.9±42.5	230, 100.5±34.8	
						TG	Current	58, 115.2±79.7	230, 97.5±70.9	
Maes M et al,1997	Cross-sectional	Belgium (Europe)	10 male, 0 female	NA	Inpatients	HDL-C	Lifetime	5, 1268.1±282.2	5, 1573.5±313.2	2
Messaoud A et at,2017	Case-control	Tunisa (Europe)	104 male, 209 female	29.84±8.78(SA), 44.33±10.5(NSA)	Outpatients	TC	Current	^c^52, 134.2±36.7 ^a^ ^c^19, 138.8±35.6 ^b^ ^c^33, 131.4±37.5	^c^110,165.1±29.0 ^a^ ^c^35, 154.6±30.5 ^b^ ^c^75, 163.2±28.2	9
						HDL-C	Current	^a^ ^c^19, 42.5±10.8 ^b^ ^c^33,39.1±15.9	^a^ ^c^35, 39.1±8.9 ^b^ ^c^75,37.9±8.19	
						LDL-C	Current	^a^ ^c^19, 91.2±39.1 ^b^ ^c^33, 79.6±42.9	^a^ ^c^35, 83.9±41.4 ^b^ ^c^75, 83.5±42.9	
						TG	Current	^a^ ^c^19, 146.2±36.3 ^b^ ^c^33, 148.0±52.3	^a^ ^c^35, 148.0±53.2 ^b^ ^c^75, 143.5±52.3	
Modai I et al,1994	Cross-sectional	Israel (Asia)	Combined	53.1(SA), 52.1(NSA)	Inpatients	TC	Current	55,207.3±39.8	106, 229±47.0	4
Olie E et al,2011	Case-control	France (Europe)	Combined	male:36.2±16.4(SA)/41.1±13.5(NSA); female:37±15(SA)/44.1±15.2(NSA)	Inpatients	TC	Lifetime	^a^148, 178±36 ^b^362, 188±37	^a^86, 217±43 ^b^189, 227±42	^7^
						TG	Lifetime	^a^148, 132±70 ^b^362, 111±53	^a^86, 150±88 ^b^189, 119±64	
Park S et al,2013	Cross-sectional	Korea (Asia)	86 male, 87female	51.6±15.1(SA), 53.2±14.8(NSA)	Inpatients	TC	Lifetime	67, 180.9±40	134,180.6±35.4	4
						HDL-C	Lifetime	67, 49.5±15.5	134,50.4±15.9	
						TG	Lifetime	67,107.8±57.9	134,131±74.1	
Peng R et al,2018	Cross-sectional	China (Asia)	371 male, 0 female	36.4±15.5 (SA), 36.4±15.7 (NSA)	Outpatients	TC	Current	69, 139.2±27.1	202, 158.5±34.8	4
						HDL-C	Current	69, 46.4±11.6	202, 46.4±11.6	
						LDL-C	Current	69, 85.1±27.1	202, 85.1±27.1	
						TG	Current	69, 79.7±35.4	202, 115.2±79.7	
Plana T et al,2010	Case-control	Spain (Europe)	33 male, 88female	15.44±1.99 (SA), 15.19±1.68 (NSA)	Inpatients	TC	Lifetime	35,148.43±26.7	25,164.6±41.25	8
Rabe-Jabłonska J et al,2000	Cross-sectional	Poland (Europe)	31 male, 71female	Not reported	Inpatients+Outpatients	TC	Current(recute) Current(remission)	30, 155±24.8 30, 229±52.3	33, 237±25.6 31, 272±48.9	3
						LDL-C	Current(recute) Current(remission)	30, 92.8±26.9 30, 116±36.6	33, 137±36.2 31, 154±37.7	
Ruljancic N et al,2011	Case-control	Crotia (Europe)	104 male, 125 female	22-57(SA), 20–59 (NSA)	Inpatients	TC	Current	^c^55,188.3±42.5	^c^77,214.6±38.7	8
Segoviano-Mendoza M et al,2018	Case-control	Mexico (America)	52 male, 211 female	35.2±10.5(SA), 37.3±10.0 (NSA)	Inpatients	TC	Current	59,152.2±39 ^a^17,140.2±32.1 ^b^42,157.1±40.8	202, 167.9±45.1 ^a^36,164.8±48.6 ^b^171,168.5±44.4	7
						HDL-C	Current	59,152.2±39 ^a^17,140.2±32.1 ^b^42,157.1±40.8	202, 167.9±45.1 ^a^36,164.8±48.6 ^b^171,168.5±44.4	
						LDL-C	Current	59,152.2±39 ^a^17,140.2±32.1 ^b^42,157.1±40.8	202, 167.9±45.1 ^a^36,164.8±48.6 ^b^171,168.5±44.4	
						TG	Current	59,172.8±88.1 ^a^17,157.6±60.2 ^b^42,179±97.2	202, 208.3±119.7 ^a^36,277.1±167.2 ^b^171,193.7±101.8	
Sullivan PF et al,1994	Cross-sectional	New Zealand (Oceania)	39 male, 51female	25.5±8.9 (SA), 35.8±10.8(NSA)	Outpatients	TC	Current	^c^13,162.0±23.9	^c^39,197.2±41.8	4
						TG	Current	^c^13,48.3±23.6	^c^39,41.4±17.0	
Su M et al, 2019	Case-control	China (Asia)	116 male, 260 female	38.76 ± 15.53 (SI), 37.13 ± 17.04 (NSI)	Inpatients+Outpatients	TC	Current	287, 177.686±40.879	89, 179.769±33.634	4
						HDL-C		287, 84.700±60.056	89, 45.232±10.835	
						LDL-C		287, 108.277±32.188	89, 111.727±28.995	
						TG		287, 51.363±41.569	89, 49.871±23.196	
Xu M et al,2006	Case-control	China (Asia)	55 male, 40 female	43±14.2	Inpatients	TC	Current	17, 125.6±15.5	27, 151.9±28.6	6
Yu C et al,2008	Case-control	China (Asia)	Combined	39.8±12.6(18–60)	Inpatients+Outpatients	TC	Current	26, 141.9±34.4	32, 169.3±42.2	7
Zhang W et al,2012	Case-control	China (Asia)	44 male, 46 female	40.3(SA)/40.7(without SA)	Inpatients	TC	Lifetime	29, 107.1±21.3	31, 151.5±22.4	6
						HDL-C		29, 38.3±8.1	31, 50.3±15.1	
						LDL-C		29, 66.1±17.4	31, 105.5±17.01	
						TG		29, 124.0±55.8	31, 127.6±52.3	
Zhao H et al, 2000	Case-control	China (Asia)	0 male, 76 female	39.81±12.63(18–60)	Inpatients	TC	Current	40, 153.5±38.3	36, 173.2±39.4	8

TC: total cholesterol. HDL-C: high-density lipoprotein-cholesterol. LDL-C: low-density lipoprotein cholesterol. TG: triglycerides. SI: suicidal ideation. SA: suicide attempt. SB: suicidal behaviour. NA: not available; NC: normal controls.

^a^ for males

^b^ for females.

^c^ for ‘mmol/l’ of the unit.

#### Serum TC concentrations and attempted suicide

**Fig 2:**
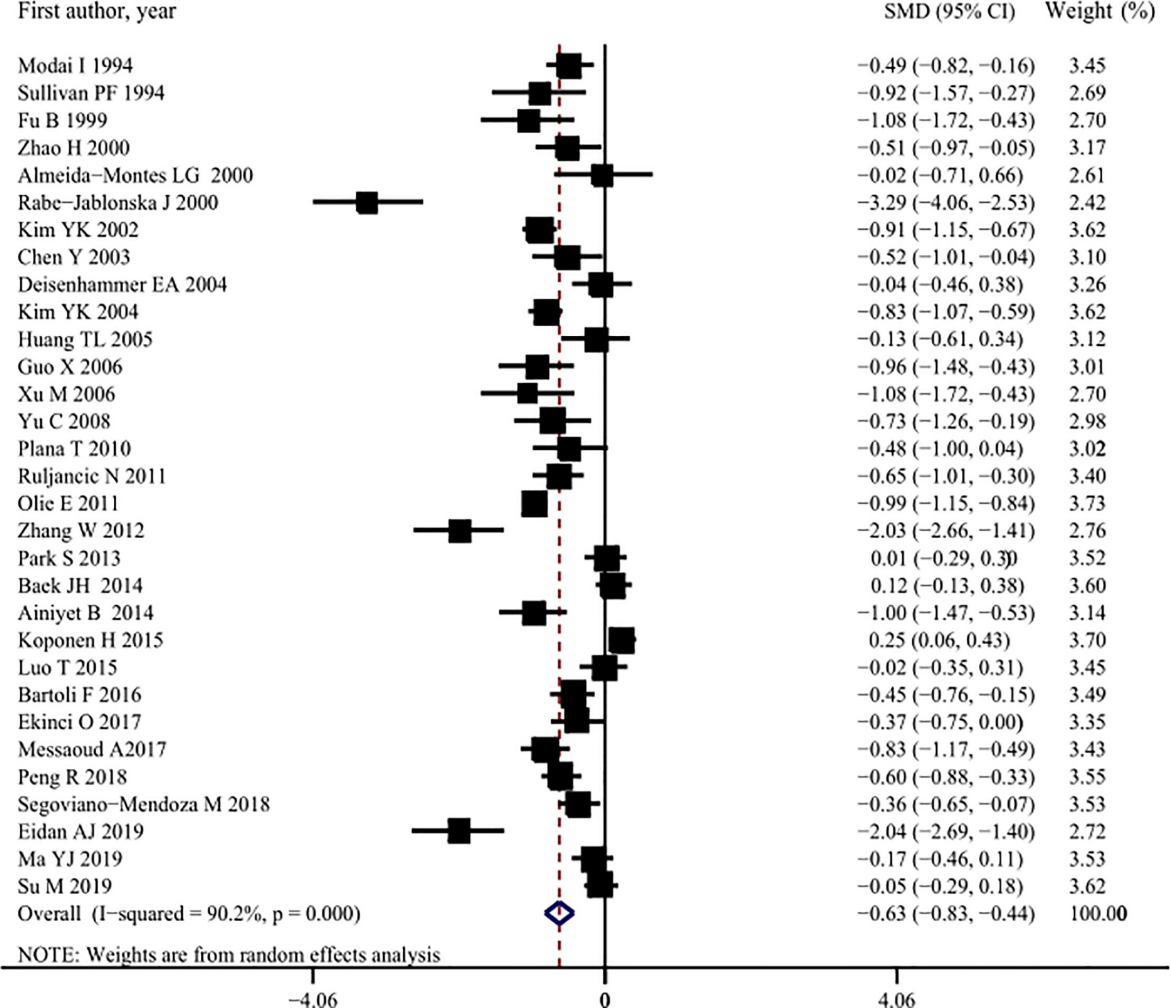
Pooled summary estimates of serum total cholesterol (TC) levels in suicide attempt versus non-suicide attempt.

Thirty-one studies [3, 7, 9, 10, 13, 15, 17–21, 31–44], comprising a total of 6,775 patients with MDD, provided data on the serum TC concentrations of suicide attempters (2,560) and non-attempters (4,227) with MDD. The serum TC concentrations of suicide attempters with MDD were significantly lower than those of non-attempters (SMD: -0.63, 95% CI: -0.83 to -0.44, *p* < 0.001) (Fig 2, Table 2). There was significant heterogeneity in the pooled analysis (*I*^2^ = 90.2%, *p* < 0.001) (Table 2). Subgroup and meta-regression analyses indicated that age (*p* = 0.041) and sample size (*p* = 0.016) might have contributed to this heterogeneity (Table 3). The sensitivity analysis did not identify any study that had affected the overall results significantly more than other studies (S1 Fig). Visual inspection of funnel plots showed that there was slight asymmetry and a statistically significant risk of publication bias (Begg’s bias: *p* = 0.022; Egger’s bias: *p* = 0.116, respectively). However, after imputing five missing studies using the trim-and-fill method, the recalculated pooled estimates were not substantially different from the initial estimates (SMD: -0.79, 95% CI: -1.01 to -0.56, *p* < 0.001) (S2 Fig).

**Table 2:** Overall and subgroup analysis for associations between serum lipid levels and suicide attempt among patients with major depressive disorder.

	TC			HDL-C			LDL-C			TG		
	n	Pooled SMD (95%CI)	*I*^*2*^	n	Pooled SMD (95%CI)	*I*^*2*^	n	Pooled SMD (95%CI)	*I*^*2*^	n	Pooled SMD (95%CI)	*I*^*2*^
**Overall**	31	-0.63 (-0.83, -0.44)	90.2	19	-0.12 (-0.33, 0.10)	85.6	18	-0.69 (-1.04, -0.34)	94.2	20	0.00 (-0.20, 0.20)	86.9
**Mean age, years**												
** <40**	12	-0.71(-0.93, -0.49)	76.3	7	0.15 (0.02, 0.28)	0.0	7	-0.34 (-0.71, 0.03)	86.1	9	-0.18 (-0.32, -0.04)	36.9
** ≥40**	18	-0.47 (-0.69, -0.24)	87.7	11	-0.24 (-0.58, 0.10)	91.0	10	-0.85 (-1.41, -0.28)	96.1	11	0.11 (-0.23, 0.44)	91.3
**Gender**												
** Female**	6	-0.69 (-1.06, -0.32)	77.8	4	0.06 (-0.16, 0.28)	0.0	4	-0.23 (-0.46, 0.01)	10.8	5	-0.12 (-0.26, 0.02)	0.0
** Male**	6	-0.81 (-1.09, -0.53)	25.7	4	0.21 (-0.12, 0.55)	0.0	4	-0.22 (-0.80, 0.36)	62.8	5	-0.43 (-0.75, -0.10)	39.8
**Geographical area**												
** America**	2	-0.31 (-0.58, -0.04)	0.0	3	0.19 (0.01, 0.38)	0.0	3	-0.10 (-0.28, 0.08)	0.0	3	-0.20 (-0.40, -0.01)	5.6
** Asia**	19	-0.60 (-0.83, -0.37)	86.8	11	-0.21 (-0.57, 0.15)	90.9	10	-1.02 (-1.62, -0.42)	95.7	10	0.08 (-0.34, 0.49)	92.5
** Europe**	9	-0.78 (-1.24, -0.31)	95.1	5	-0.07 (-0.33, 0.19)	56.6	5	0.37 (-0.93, 0.20)	92.2	6	-0.09 (-0.28, 0.11)	68.2
** Oceania**	1	-0.92 (-1.57, -0.27)	-	-	-	-	-	-	-	1	0.53 (-0.10, 1.17)	-
**Setting**												
** Inpatients**	19	-0.56 (-0.77, -0.35)	83.1	12	-0.32 (-0.61, -0.03)	83.3	10	-0.91 (-1.48, -0.33)	94.8	12	0.01 (-0.31, 0.32)	91.0
** Outpatients**	8	-0.68 (-1.12, -0.24)	94.5	5	0.06 (-0.12, 0.25)	51.3	5	-0.28 (-0.76, 0.20)	92.8	6	-0.05 (-0.32, 0.22)	76.8
** Combined**	4	-0.99 (-2.18, 0.20)	95.4	2	0.52 (-0.07, 1.11)	64.8	3	-0.62 (-1.43, 0.20)	88.4	2	0.06 (-0.17, 0.29)	0.0
**Sample size**												
** <100**	13	-1.03 (-1.47, -0.59)	87.1	8	-0.22 (-0.53, 0.09)	62.7	8	-1.22 (-1.80, -0.65)	88.5	6	-0.12 (-0.40, 0.17)	41.3
** ≥100**	18	-0.41 (-0.63, -0.20)	91.0	11	-0.06 (-0.33, 0.22)	90.3	10	-0.29 (0.66, 0.08)	93.9	14	0.04 (-0.20, 0.27)	90.4
**Study design**												
** Case-control**	18	-0.76 (-0.97, -0.54)	84.6	9	-0.22 (-0.69, 0.25)	92.9	9	-1.13 (-1.79, -0.46)	96.1	9	0.13 (-0.26, 0.52)	92.9
** Cross-sectional**	13	-0.45 (-0.76, -0.14)	90.3	10	-0.01 (-0.14, 0.11)	29.9	9	-0.25 (-0.55, 0.05)	85.3	11	-0.11 (-0.29, 0.07)	68.4
**Publication year**												
** Before 2007**	14	-0.77 (-1.04, -0.49)	82.3	7	-0.24 (-0.60, 0.12)	67.0	6	-0.74 (-1.32, -0.16)	86.2	5	-0.12 (-0.46, 0.23)	52.0
** 2007 or later**	17	-0.53 (-0.80, -0.27)	92.4	12	-0.06 (-0.32, 0.21)	89.4	12	-0.66 (1.09, -0.24)	95.4	15	0.03 (-0.20, 0.26)	89.7
**Suicidal time-frame**												
** Recent**	21	-0.73 (-0.94, -0.52)	84.2	11	-0.06 (-0.40, 0.28)	89.5	12	-0.84 (-1.31, -0.37)	94.4	12	0.11 (-0.24, 0.46)	90.9
** Lifetime**	10	-0.43 (-0.83, -0.03)	94.5	8	-0.17 (-0.41, 0.07)	72.8	6	-0.38 (-0.88, 0.12)	92.7	8	-0.11 (-0.29, 0.07)	68.7
**Blood sample**												
** Fasting**	24	-0.56 (-0.77, -0.35)	90.3	16	-0.11 (-0.36, 0.14)	87.7	15	-0.56 (-0.93, -0.20)	94.2	17	0.07 (-0.15, 0.29)	88.0
** Non-fasting**	7	-0.96 (-1.60, -0.34)	91.2	3	-0.13 (-0.37, 0.10)	6.9	3	-1.31 (-1.90, -0.71)	71.9	3	-0.35 (-0.54, -0.16)	0.0
**Treatment**												
** Yes**	10	-0.69 (-1.09, -0.29)	92.3	7	0.05 (-0.30, 0.40)	88.4	7	-0.54 (-1.05, -0.04)	94.3	7	-0.09 (-0.30, 0.13)	68.2
** No**	21	-0.61 (-0.83, -0.39)	88.2	12	-0.23 (-0.50, 0.05)	83.1	11	-0.78 (-1.27, -0.29)	93.9	13	0.07 (-0.23, 0.36)	90.4
**Study quality**												
** Relatively high**	19	-0.49 (-0.69, -0.28)	87.4	11	-0.11 (-0.35, 0.14)	84.7	10	-0.68 (-1.18, -0.18)	95.9	12	0.09 (-0.22, 0.40)	91.5
** Relatively low**	12	-0.91 (-1.31, -0.50)	91.8	8	-0.16 (-0.61, 0.30)	87.7	8	-0.70 (-1.18, -0.22)	89.4	8	-0.11 (-0.27, 0.05)	40.6

Abbreviations: SMD, standardized mean differences

**Table 3:** The meta-regression analysis between serum lipid levels and suicide attempt among patients with major depressive disorder.

	Coefficient	Standard error	t	P value	95% CI of intercept
**TC**					
** **Mean age	0.408501	0.1913792	2.13	**0.041**	(0.0170865, 0.7999155)
** **Gender	-0.1023742	0.2444239	-0.42	0.684	(-0.6469847, 0.4422362)
** **Geographical area	-0.2036264	0.1990552	-1.02	0.315	(-0.6107399, 0.2034872)
** **Setting	-0.156357	0.1778468	-0.88	0.387	(-0.5200946, 0.2073806)
** **Sample size	0.5956776	0.2321494	2.57	**0.016**	(0.1208787, 1.070477)
** **Study design	0.3137544	0.2477391	1.27	0.215	(-0.192929, 0.8204378)
** **Publication year	0.2424042	0.2499107	0.97	0.340	(-0.2687205, 0.7535289)
** **Suicidal time-frame	0.3190186	0.260349	1.23	0.230	(-0.213455, 0.8514922)
** **Blood sample	0.3709426	0.299333	1.24	0.225	(-0.2412621, 0.9831472)
** **Treatment	-0.0635281	0.2696655	-0.24	0.815	(-0.615056, 0.4879999)
** **Study quality	-0.4000958	0.2495565	-1.60	0.120	(-0.9104962, 0.1103046)
**HDL-C**					
** **Mean age	-0.4301005	0.2082575	-2.07	0.054	(-0.8694853, 0.0092844)
** **Gender	0.1552346	0.2033184	0.76	0.474	(-0.3422675, 0.6527367)
** **Geographical area	-0.1142483	0.1920367	-0.59	0.560	(-0.5194103, 0.2909137)
** **Setting	0.4055806	0.1531858	2.65	**0.017**	(0.0823868, 0.7287744)
** **Sample size	0.1771247	0.2508928	0.71	0.490	(-0.3522128, 0.7064622)
** **Study design	0.165694	0.243013	0.68	0.505	(-0.3470187, 0.6784067)
** **Publication year	0.1918538	0.2590181	0.74	0.469	(-0.3546267, 0.7383343)
** **Suicidal time-frame	-0.1479952	0.2471545	-0.60	0.557	(-0.6694455, 0.3734552)
** **Blood sample	0.0480444	0.3378913	0.14	0.889	(-0.664844, 0.7609327)
** **Treatment	0.279337	0.2433251	1.15	0.267	(-0.234034, 0.792708)
** **Study quality	-0.033727	0.2525184	-0.13	0.895	(-0.5664942, 0.4990401)
**LDL-C**					
** **Mean age	-0.4713055	0.405141	-1.16	0.262	(-1.330166, 0.387555)
** **Gender	0.0789991	0.3092765	0.26	0.807	(-0.6777733, 0.8357714)
** **Geographical area	0.0085279	0.3685013	0.02	0.982	(-0.7726599, 0.7897156)
** **Setting	0.223375	0.3180183	0.70	0.493	(-0.4507938, 0.8975437)
** **Sample size	0.9188531	0.4325204	2.12	0.050	(0.0019509, 1.835755)
** **Study design	0.8294952	0.4382822	1.89	0.077	(-0.0996215, 1.758612)
** **Publication year	0.060802	0.5178604	0.12	0.908	(-1.037013, 1.158617)
** **Suicidal time-frame	0.4268673	0.5028153	0.85	0.408	(-0.6390535, 1.492788)
** **Blood sample	0.7293679	0.6320634	1.15	0.265	(-0.6105466, 2.069282)
** **Treatment	0.1949679	0.4952573	0.39	0.699	(-0.8549308, 1.244867)
** **Study quality	-0.0240811	0.4893844	-0.05	0.961	(-1.06153, 1.013367)
**TG**					
** **Mean age	0.2264841	0.2704247	0.84	0.413	(-0.3416571, 0.7946253)
** **Gender	-0.2237067	0.1353678	-1.65	0.137	(-0.5358655, 0.0884521)
** **Geographical area	0.0044212	0.2024676	0.02	0.983	(-0.4209476, 0.4297899)
** **Setting	0.0607395	0.1890078	0.32	0.752	(-0.336351, 0.4578301)
** **Sample size	0.1283375	0.3066807	0.42	0.681	(-0.5159748, 0.7726497)
** **Study design	-0.2243173	0.2699813	-0.83	0.417	(-0.791527, 0.3428923)
** **Publication year	0.1204467	0.3262036	0.37	0.716	(-0.5648817, 0.8057751)
** **Suicidal time-frame	-0.2415413	0.2724502	-0.89	0.387	(-0.8139379, 0.3308552)
** **Blood sample	0.4601997	0.365089	1.26	0.224	(-0.3068239, 1.227223)
** **Treatment	-0.1769625	0.2831946	-0.62	0.540	(-0.7719322, 0.4180072)
** **Study quality	-0.2146197	0.2761651	-0.78	0.447	(-0.7948211, 0.3655817)

NOS, Newcastle-Ottawa Scale.

#### Serum HDL-C concentrations and attempted suicide

**Fig 3:**
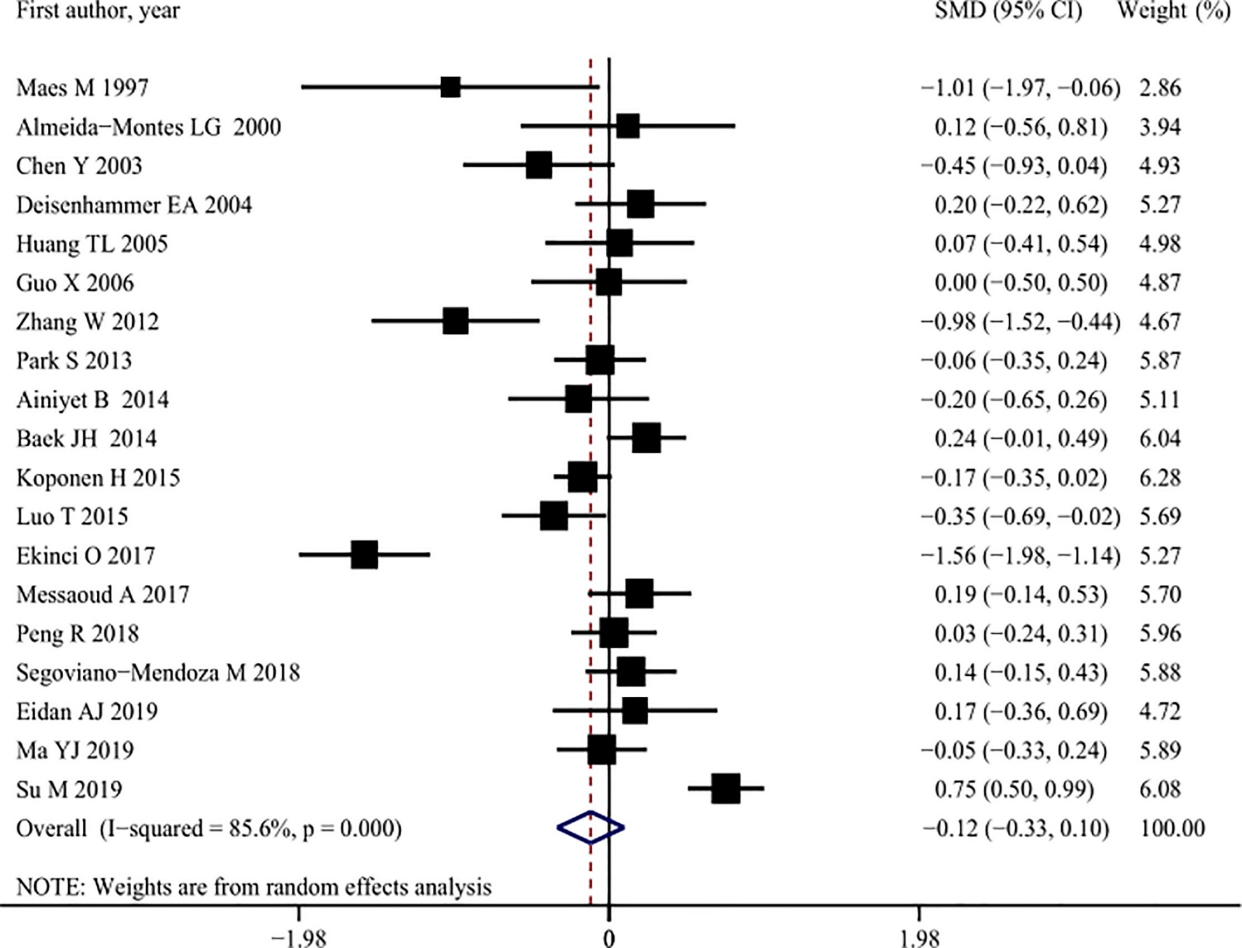
Pooled summary estimates of serum high-density lipoprotein cholesterol (HDL-C) levels in suicide attempt versus non-suicide attempt.

A meta-analysis of 19 studies [3, 9, 13, 15, 17–22, 31, 32, 35, 37, 42, 44–48], comprising a total of 4,284 patients with MDD, showed that there were no significant differences in the serum HDL-C concentrations between suicide attempters (1,309) and non-attempters (2,975) (SMD: -0.12, 95% CI: -0.33 to 0.10), and that there was significant heterogeneity among studies (*I*^2^ = 8 5.6%, *p* < 0.001) (Fig 3, Table 2). However, subgroup analysis showed that the serum HDL-C concentrations of inpatients were significantly lower among suicide attempters than among non-attempters (SMD: -0.32, 95% CI: -0.61 to -0.03) (Table 2). Meta-regression analyses confirmed that the significant heterogeneity might be partly due to different settings (*p* = 0.017) (Tables 2 and 3). Sensitivity analyses did not find any study that significantly affected the overall results more than other studies (S3 Fig). No asymmetry was found in funnel plots by visual assessment, and Begg’s and Egger’s test showed that there was no publication bias (Begg’s bias: *p* = 0.080; Egger’s bias: *p* = 0.148) (S4 Fig).

#### Serum LDL-C concentrations and attempted suicide

**Fig 4:**
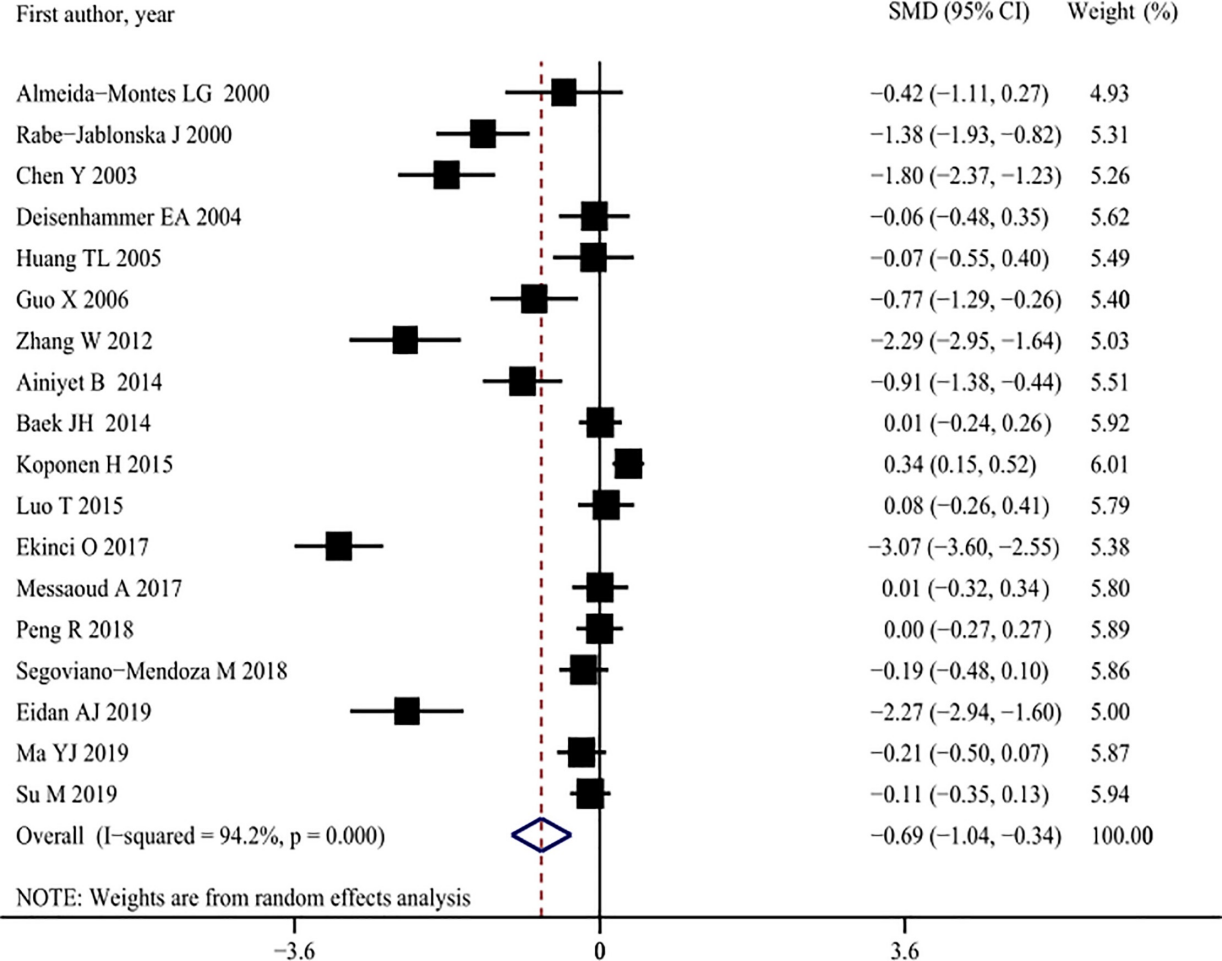
Pooled summary estimates of serum low-density lipoprotein cholesterol (LDL-C) levels in suicide attempt versus non-suicide attempt.

Seventeen studies [3, 9, 13, 15, 17–22, 31, 32, 35, 39, 42, 44, 46–48], comprising a total of 3,811 subjects, provided data on serum LDL-C concentrations. The serum LDL-C concentrations of suicide attempters (1,294) with MDD were significantly lower than those of non-attempters (2,893) (SMD: -0.69, 95% CI: -1.04 to -0.34), and there was significant heterogeneity among studies (*I*^2^ = 94.2%, *p* < 0.001) (Fig 4, Table 2). Subgroup analysis showed that significant differences were present only in studies that comprised inpatients and outpatients, comprised those who had recently attempted suicide, used cross-sectional designs, were published before 2007, non-fasting blood sample and reported relatively low-quality comparisons of suicide attempters with non-attempters, but the contributions of these factors were not confirmed in meta-regression analyses (Tables 2 and 3). The sensitivity analyses did not find any study that had affected the overall results significantly more than others (S5 Fig). Visual assessment showed that there was a partial asymmetry in the funnel plot, and Begg’s test and Egger’s test showed that there was a potential publication bias (both Begg’s and Egger’s bias: *p* < 0.001). After using the trim-and-fill method, one study was filled and the recalculated pooled estimate was found to not be significantly different (SMD: -0.76, 95% CI: -1.18 to -0.35, *p* < 0.001) (S6 Fig).

#### Serum TG concentrations and attempted suicide

**Fig 5:**
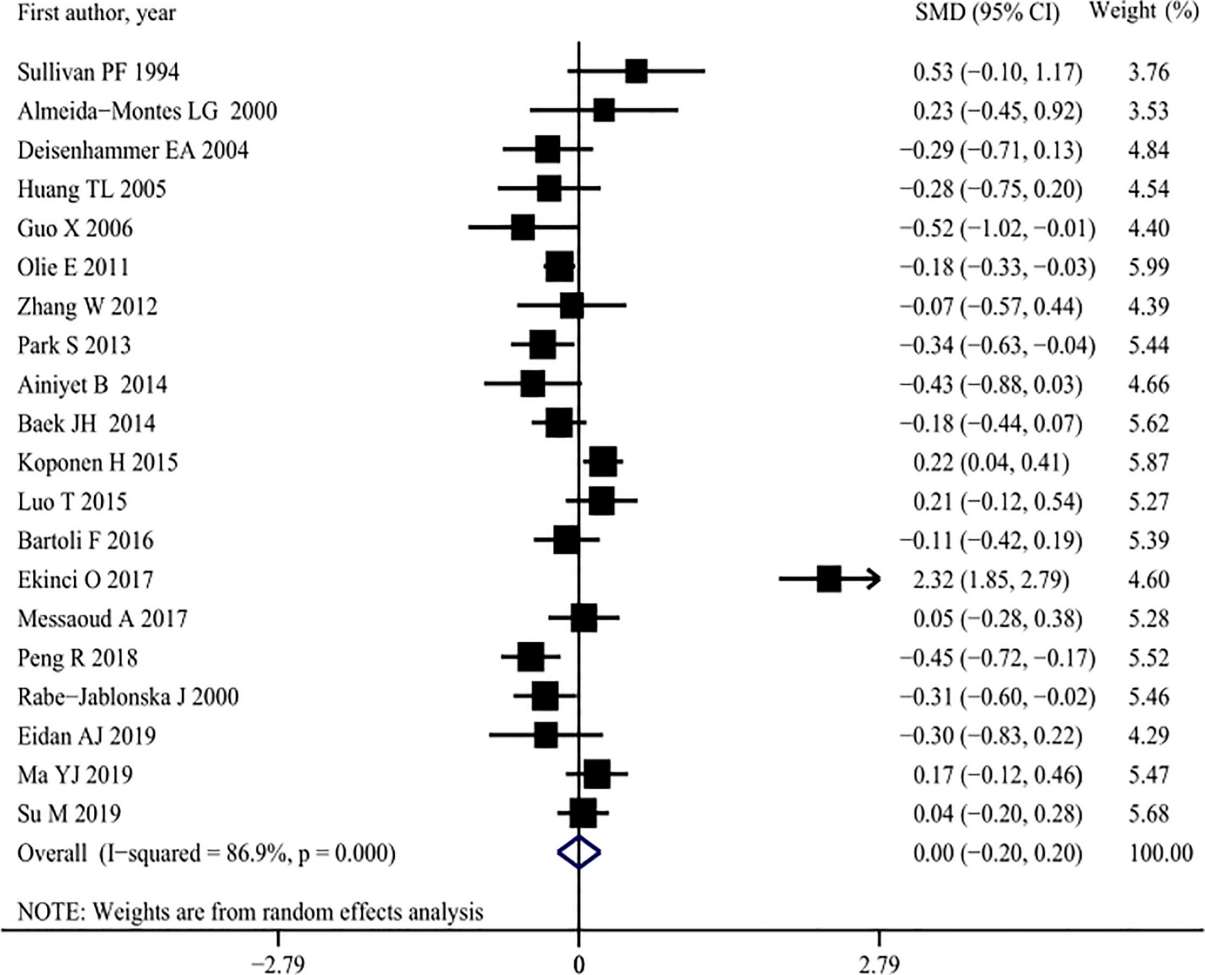
Pooled summary estimates of serum triglycerides (TG) levels in suicide attempt versus non-suicide attempt.

A meta-analysis of 19 studies [3, 7, 9, 10, 13, 15, 17–22, 31, 32, 35, 37, 41, 44, 46–48], comprising a total of 5,211 patients with MDD, showed that there were no significant differences in serum TG concentrations between suicide attempters (1,857) and non-attempters (3,354) (SMD: 0.00, 95% CI: -0.20 to 0.20), and that there was significant heterogeneity among studies (*I*^2^ = 86.9%, *p* < 0.001) (Fig 5, Table 2). Subgroup analysis confirmed these results, which suggested that there was a significant effect of low TG concentrations on attempted suicide (Table 2), although studies comprising younger participants (< 40 years old) or those that had been performed in the US had relatively low quality. However, no contribution from these factors was observed in meta-regression analyses (Table 3). The sensitivity analyses did not find that any study had significantly affected the overall results more than other studies (S7 Fig). No asymmetry was found in the funnel plot by visual assessment, and Begg’s and Egger’s test showed that there was no publication bias (Begg’s bias: *p* = 0.347; Egger’s bias: *p* = 0.534) (S8 Fig).

## Discussion

This was a comprehensive meta-analysis of 32 observational studies and involved a pooled analysis. It comprised a total of 2,568 suicide attempters and 4,407 non-attempters with MDD, and assessed the associations between serum lipid concentrations and attempted suicide in these patients. It was found that suicide attempters with MDD had significantly lower serum TC and LDL-C concentrations than non-attempters with MDD, but that there were no significant differences in the serum HDL-C and TG concentrations between these groups. These findings support the hypothesis that lower serum TC and LDL-C concentrations are associated with attempted suicide in MDD patients.

The association between serum lipid concentrations and attempted suicide in MDD patients remains controversial. A majority of studies have suggested that lower serum cholesterol concentrations may be associated with suicide attempt in MDD patients [3, 10, 31, 33–36, 38–44, 46, 48–52]. Seven other studies [17, 18, 31, 39, 42, 46, 48] have found that suicide attempters with MDD had significantly lower LDL-C concentrations than non-attempters with MDD. A recent meta-analysis [16] consistently found an inverse association between lipid concentrations and suicidality. This study not only found that patients with suicidal attempt had lower serum TC and TG levels than non-suicidal patients, but also reported that lower serum TC, HDL-C, LDL-C concentrations among patients who attempted to commit suicide compared with the healthy participants. However, the meta-analysis by Wu et al. [16] was conducted among patients with various psychiatric diseases, and they had combined the data from patients with various types of depressive orders (depression, chizoaffective depression, major depressive episodes and MDD) rather than focusing on MDD patients, this might lead to heterogeneity between patients with different kinds of depressive disorders. Moreover, compared with that 32 eligible studies based on MDD patients were included in our study, only 18 studies of suicidal attempter with depression were finally analysed in their study. The heterogeneity of studied populations and the gap of sample size might partly explain the inconsistent results between their study and the present paper.

Our results were consistent with the above studies’ findings, which suggests that low serum lipid concentrations in an MDD patient may be a predictive biomarker for attempted suicide. Additionally, our study also found that there were no significant differences in the serum TG and HDL-C concentrations between MDD patients who did attempted suicide and those who did not. However, Koponen *et al*. [35] showed that MDD patients who attempted suicide had significantly higher TC, LDL-C and TG concentrations than non-suicide attempters, and that disturbances in glucose metabolism were associated with attempted suicide. Moreover, some studies have suggested that low serum TG and HDL-C concentrations were associated with attempted suicide in MDD patients [3, 9, 10, 31, 35, 36, 41]. However, Baek *et al*. [9] found that MDD patients who attempted suicide had significantly higher HDL-C concentrations than those who did not. These results were opposite to ours, which may be partially ascribable to methodological limitations such as small sample sizes, wide inter-study variation in the definitions or classification schemes of attempted suicide, the types of serum lipid measured and the concentration assessment used, and confounding factors, such as obesity.

Our study showed that there was a significant association between low serum TC and LDL-C concentrations and attempted suicide in MDD patients. Despite the significant heterogeneity across studies, this finding was reliable due to the larger sample size and inclusion of a broad range of studies. In addition, our subgroup analyses revealed that the studies’ characteristics did not have a significant influence on the inverse association between serum TC and LDL-C concentrations and attempted suicide in MDD patients. Interestingly, there was a stronger association between lower serum TC concentrations and attempted suicide in middle- and older-aged patients than in those younger than 40 years, which is consistent with a previous study [53]. This may be due to the age-related increase in somatic diseases and decline of cognitive abilities, or the fact that older people are more likely to live alone and suffer economic hardship.

Our meta-analysis also showed that recent suicide attempters with MDD had significantly lower serum TC and LDL-C concentrations than those who had not recently attempted suicide, but Baek et al. [9] found no significant differences in the serum TC and LDL-C concentrations between those who had recently attempted suicide and those who had not. In addition, long-term prospective studies have only observed an association between low cholesterol concentrations and suicidality during the early years of follow-up [31]. This may be due to measurement limitations and sample-selection differences across studies. Overall, our results support the hypothesis that lower serum TC and LDL-C concentrations are associated with attempted suicide in MDD patients.

The mechanisms that link serum lipid concentrations with attempted suicide in MDD patients are not fully understood. Several theories have been postulated, such as that lower peripheral lipid concentrations may change the viscosity of membrane lipids in brain cells, thereby affecting synaptic plasticity and causing general brain dysfunction [3]. Previous studies have indicated that serum cholesterol concentrations were positively correlated with the concentrations of 5-hydroxyindoleacetic acid (the primary metabolite of serotonin) in the cerebrospinal fluid, which is associated with the risk of suicide [15, 54]. Moreover, the association between lipids and serotonin may have a genetic background, as there appears to be a link in some depressed patients with the short allele of the serotonin transporter gene polymorphism and lower LDL concentrations, which may reflect the effect of synaptic plasticity on brain dysfunction [5, 55]. Some studies have shown that the relationship of serum cholesterol concentrations and suicidality in patients with depression was correlated with interleukin-2, and manifested as lower TC and higher TG concentrations [56, 57], while other have found that lipid-lowering drugs may have an antidepressant effect via anti-inflammatory pathways [9, 58]. Other researchers have suggested that polyunsaturated fatty acid concentrations or the balance of omega-3 and omega-6 polyunsaturated fatty acids may play an important role in serotonergic function in suicidality [12, 59, 60]. Specifically, a lower total concentration of omega-3 fatty acids and an increased omega-6 to omega-3 ratio may disrupt the biophysical properties of the neuronal membrane, thereby influencing serotonin uptake, the binding of b2 adrenergic and serotonergic receptors to their respective ligands, and monoamine oxidase activity, all of which have been linked to MDD [61].

There are several limitations to our study. First, many other factors that may influence serum lipid concentrations were not included in our subgroup analyses, such as genetic factors, cigarette smoking, alcohol consumption, dietary habits, physical activity and comorbid physical conditions. Second, although random-model, subgroup and meta-regression analyses were performed, heterogeneity was unavoidable in most of the analyses; this heterogeneity may be partly attributable to variations in the ages of study participants, sample sizes, and study settings. In addition, the relatively low quality of some studies (which were unadjusted, or adjusted only for a few important factors) may have diminished the meta-analytical comparability of groups on the basis of study design or analysis. The relationships we found were highly consistent, although the eligible studies were heterogeneous. Moreover, as observational studies were included in the meta-analysis, the cause-and-effect association between serum lipid concentrations and attempted suicide in MDD patients is unclear. In addition, due to a lack of information we could not examine the associations between lipid concentrations and the type of suicide attempt; future work should explore whether attempted violent suicide is predicted by low serum lipid concentrations.

## Conclusion

The results of our meta-analysis suggested that MDD patients with who attempted suicide had significantly lower serum TC and LDL-C concentrations than those who did not, but no difference was found in the serum HDL-C and TG concentrations between these groups. Lower serum TC and LDL-C concentrations may thus be predictive biomarkers of attempted suicide in MDD patients.

## Supporting information

S1 Checklist PRISMA 2009 checklist. (DOC)

S1 Fig Sensitivity analysis of serum TC levels and suicide attempt in MDD. (DOCX)

S2 Fig Funnel plot of publication bias in serum TC levels and suicide attempt in MDD. (DOCX)

S3 Fig Sensitivity analysis of serum HDL-C levels and suicide attempt in MDD. (DOCX)

S4 Fig Funnel plot of publication bias in serum HDL-C levels and suicide attempt in MDD. (DOCX)

S5 Fig Sensitivity analysis of serum LDL-C levels and suicide attempt in MDD. (DOCX)

S6 Fig Funnel plot of publication bias in serum LDL-C levels and suicide attempt in MDD. (DOCX)

S7 Fig Sensitivity analysis of serum TG levels and suicide attempt in MDD. (DOCX)

S8 Fig Funnel plot of publication bias in serum TG levels and suicide attempt in MDD. (DOCX)
