# Supplementary figures and images for: Association between serum lipid concentrations and attempted suicide in patients with major depressive disorder: A meta-analysis
Source: PLoS One. 2020 Dec 10;15(12):e0243847. doi: 10.1371/journal.pone.0243847 (PMC7728216; doi:10.1371/journal.pone.0243847)

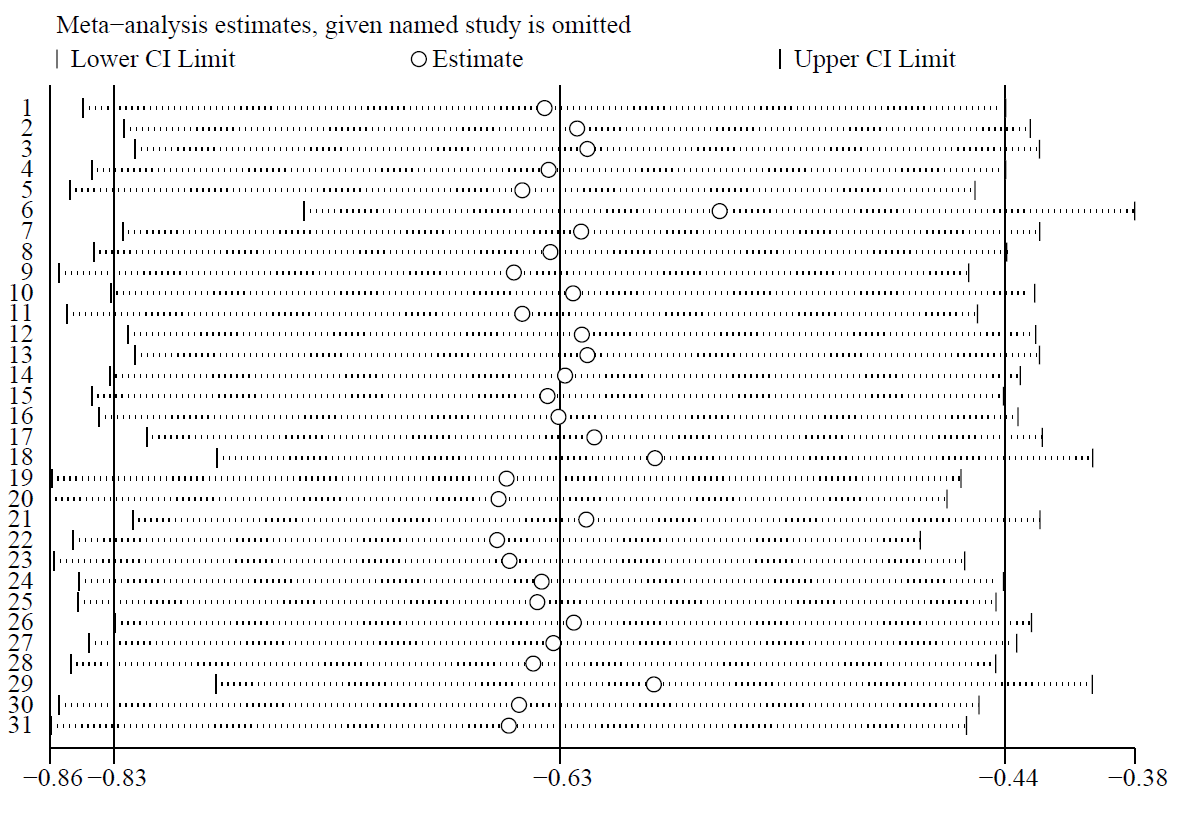


**S1 Fig. Sensitivity analysis of serum TC levels and suicide attempt in MDD.**

Supplement: S1 Fig — (DOCX) [file pone.0243847.s002.docx]

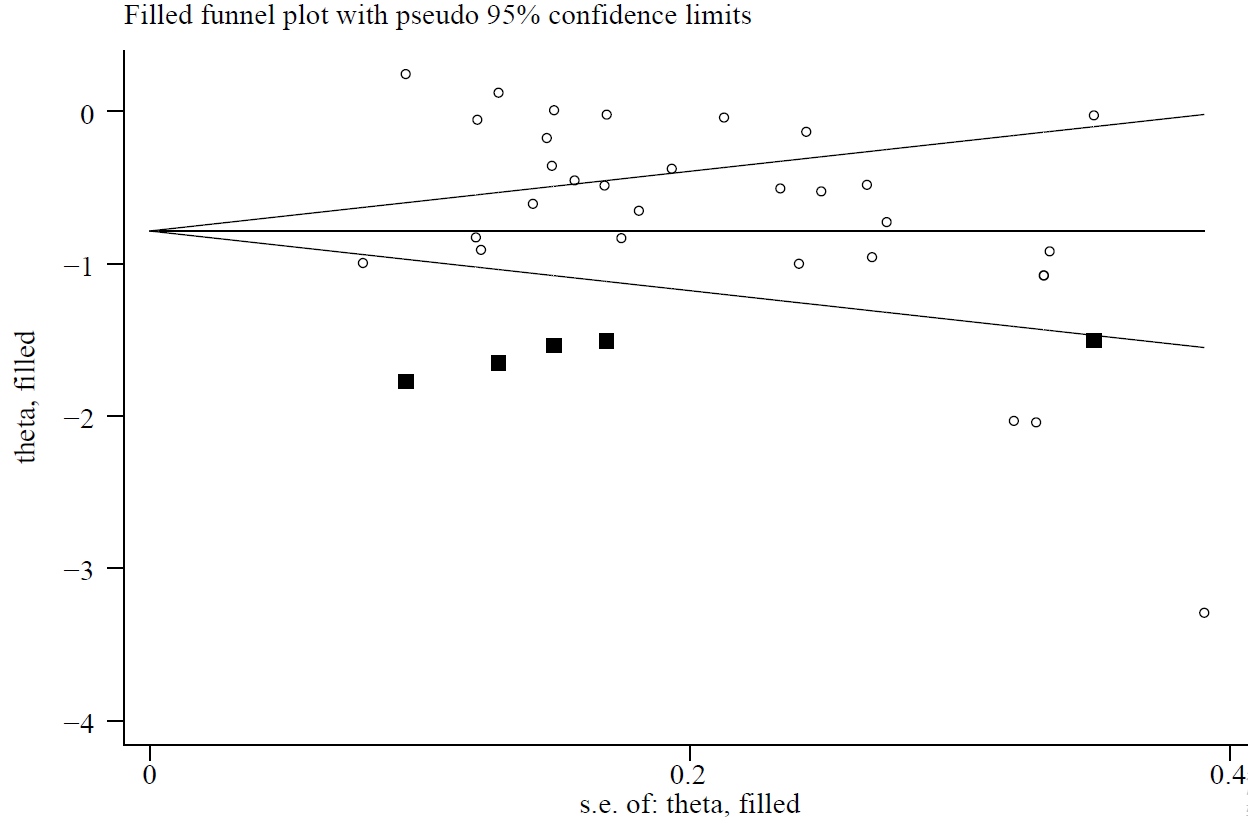


**S2 Fig. Funnel plot of publication bias in serum TC levels and suicide attempt in MDD.**

Supplement: S2 Fig — (DOCX) [file pone.0243847.s003.docx]

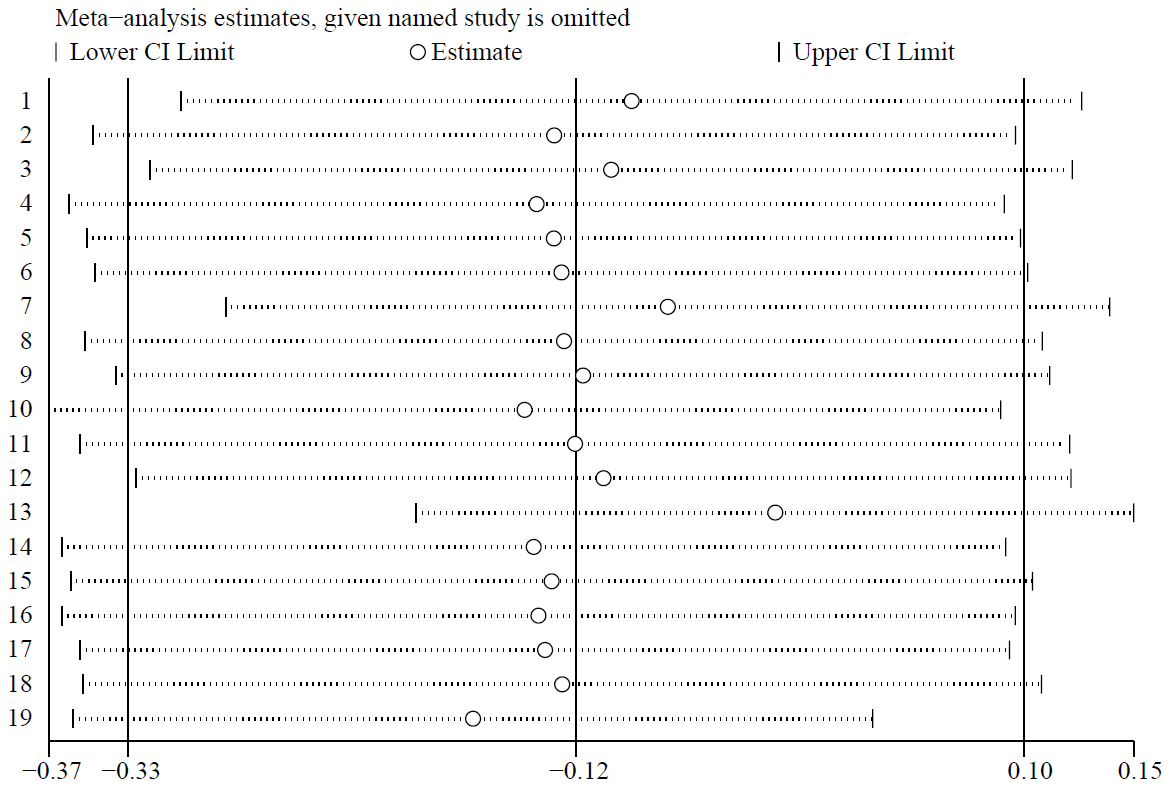


**S3 Fig. Sensitivity analysis of serum HDL-C levels and suicide attempt in MDD.**

Supplement: S3 Fig — (DOCX) [file pone.0243847.s004.docx]

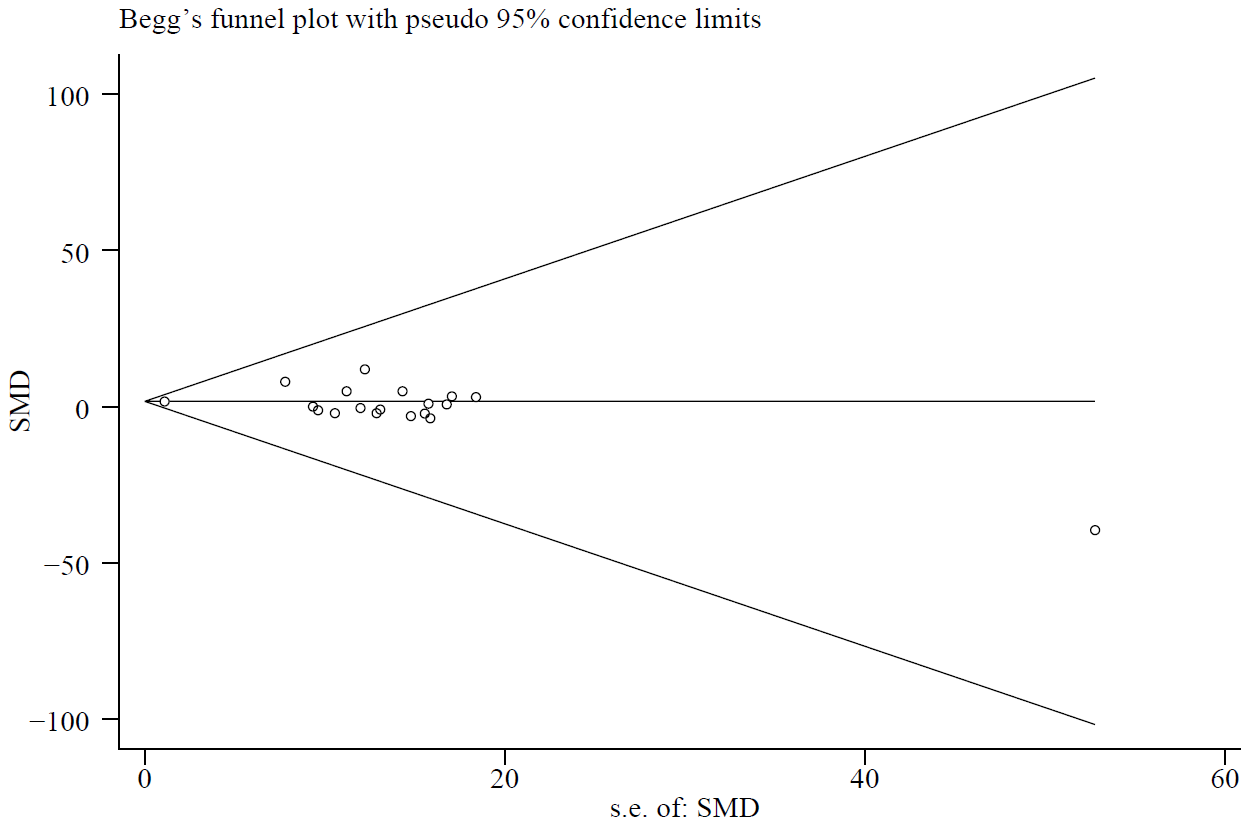


**S4 Fig. Funnel plot of publication bias in serum HDL-C levels and suicide attempt in MDD.**

Supplement: S4 Fig — (DOCX) [file pone.0243847.s005.docx]

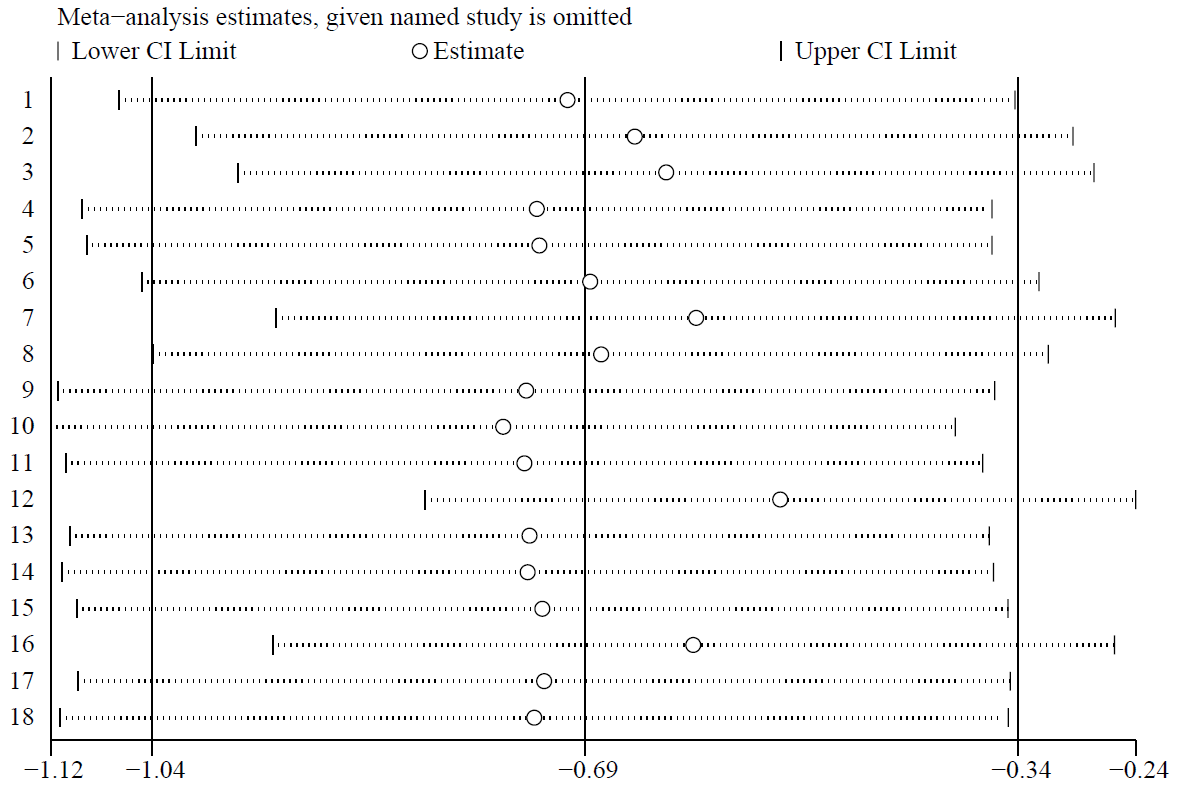


**S5 Fig. Sensitivity analysis of serum LDL-C levels and suicide attempt in MDD.**

Supplement: S5 Fig — (DOCX) [file pone.0243847.s006.docx]

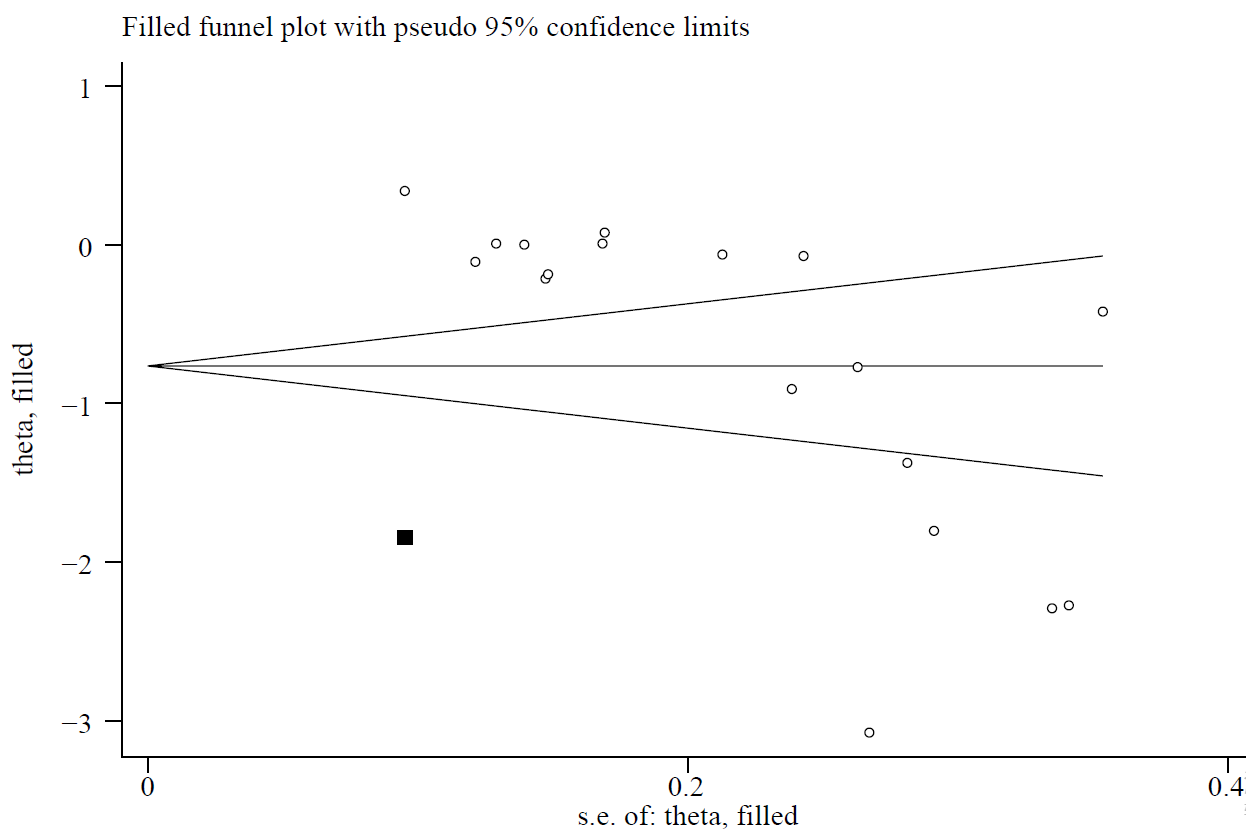


**S6 Fig. Funnel plot of publication bias in serum LDL-C levels and suicide attempt in MDD.**

Supplement: S6 Fig — (DOCX) [file pone.0243847.s007.docx]

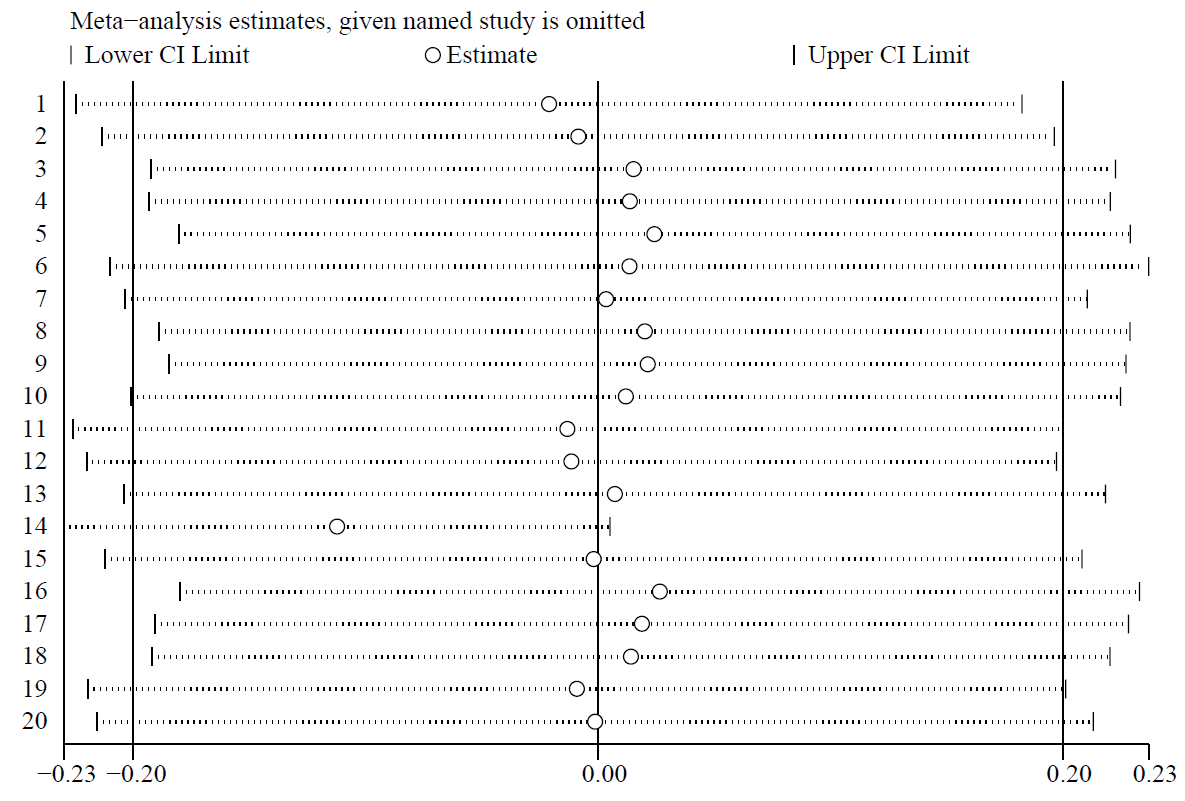


**S7 Fig. Sensitivity analysis of serum TG levels and suicide attempt in MDD.**

Supplement: S7 Fig — (DOCX) [file pone.0243847.s008.docx]

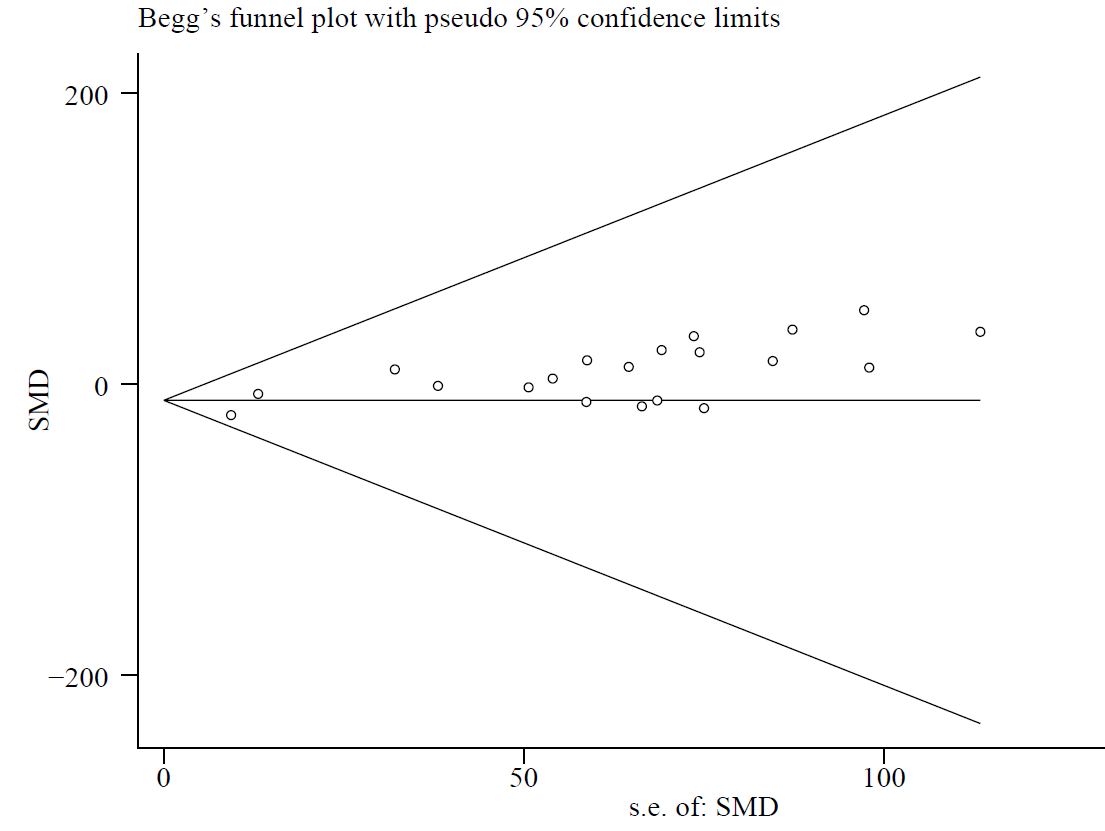


**S8 Fig. Funnel plot of publication bias in serum TG levels and suicide attempt in MDD.**

Supplement: S8 Fig — (DOCX) [file pone.0243847.s009.docx]
